# Annotations

**Published:** 1889-02-16

**Authors:** 


### ANNOTATIONS.

**"East-Wind" Liver.**

THE east wind season is approaching, and many persons begin to anticipate inconvenience from it. It is an enemy to many, a friend to very few. Those who suffer from chest complaints generally make preparation beforehand, in anticipation of the enemy's advent. The unfortunates whose livers are disturbed by the east wind are often quite unaware of the cause of their troubles, and when they do know it, are sometimes at a loss as to what may be the best preventive of mischief. Undoubtedly, the best of all preventives is a robust state of health. When the health is robust all the physiological processes go on regularly and merrily, like the work of a mill situated on the bank of a full stream. But if the health get down, the liver, in certain persons, is sure to suffer at the first approach of east wind. Regular living, plenty of exercise and fresh air are by far the best methods of preparing for the approach of March and April. But the winter has been unfavourable for outdoor pleasures, and it is to be feared that many people are in consequence a little "below par," and not braced up for the coming season. For such there is no time to be lost. The exercise, the regular meals, the fresh air, should all be invoked with the utmost promptitude. One other defence ought not to be forgotten. Warm woollen underclothing is as essential for those whose livers are deranged by the cold as for those who have unsatisfactory chests. This clothing should be sufficiently thick and sufficiently long. It should, in fact, extend from throat to foot. A flannel bandage round the abdomen, extending above the level of the liver, is also necessary for some persons. Warm, thick boots and stockings likewise are not to be omitted. It is better to take these precautions promptly than to endure the feeling of stupidity, headache, nausea, irritability of temper, and the other worries innumerable which accompany an attack of "liver." Such an attack is not usually dangerous, but it is very disabling and misery-producing, and may be the precursor of much more serious troubles. Prevention is possible, but only to those who have sufficient energy of character to carry out the ordinary rules of health with regularity.

**Football Brutalities.**

BRITISH pluck is an endowment of which any nation might be justly proud. The robust energy of our schoolboys and young men, and their preference for games which demand strenuousness, boldness, and invincible determation, are as honourable to themselves as they are undoubtedly extraordinary. But much as we admire the sight of two cricket teams doing cool and resolute battle in the summer, or the picked men of two football clubs engaged in a hand-to-hand and foot-to-foot struggle, as if for dear life, in the winter, we cannot shut our eyes to such facts as have been forced upon public attention since the present year commenced. During one week no fewer than seven accidents of a serious and dangerous character were reported, two of which have since proved fatal. Cropper, a noted player, was killed at Grimsby; Beasley sustained concussion of the brain at Leicester; Black, at Stoneywood, near Aberdeen, had his leg broken; Grey, at Chelmsford, had a leg broken; Thomas, at Gloucester, is reported with a fractured thigh; Moir, of Masborough, near Sheffield, with a ruptured kidney; and Smith, of Charlton, killed. All these major casualties within one week! How many minor accidents have occurred no one knows: how many serious ones in districts where newspaper reporters are unknown. But these are enough. Whatever that portion of the public who have no sons may think, whatever schoolmasters may say, there is no doubt that those who sorrow with their injured living, and those who mourn their premature dead, will regard football with feelings of hatred and horror. Every parent who has boys at school, or in business will dread the approach of days on which matches are to be played. Is it just or humane that all the parents in the country should be compelled to bear months of agonising suspense during every football season? It is not to be endured. Accidents and casualties there must be in life, and all games cannot be prohibited because there is an element of risk in some. But is it not possible to play football with the keenest enjoyment, with the sternest sense of conflict, with the utmost possible strenuousness without such risks as now attend the game? Every experienced player in the kingdom knows that it is. The rules of the game must be reconsidered. The authority of "Rugby" and of the "Association" must come to an end. The best brains of the football world should meet in conference and consider the question from every point of view. One of two things is certain; either the rules must be modified, or the game itself must be discontinued. Sorrowing and broken-hearted parents will not endure it. The discontinuance of the game would be a national misfortune; but its continuance in its present form is a national calamity. We urge upon all players the necessity for immediate and decisive action, and upon all parents, head masters, and other responsible persons, the danger and scandal of the present brutal game.

**Are Abstainers Long-lived?**

ABSTAINERS and life assurance offices have decided this question in the affirmative. But the present age is nothing if not critical, and "all things, both great and small," are put again and again through the mill of criticism without fear and without remorse. To the inexperienced it seems as if it ought to be perfectly easy to decide a point of this kind, and to make the evidence so plain and clear that even opponents should feel constrained to close their mouths. But the initial difficulties of an inquiry into the longevity of any class are very great, and when the inquiry has been made and completed, it is so difficult as to be practically impossible to get the conclusions universally made known, much more to get them universally believed. Questions that are settled, and have been settled for ages among the initiated, are constantly raised afresh by the ignorant, and often make no small stir among people who are like-minded. For example, there are not wanting even educated men who persist in arguing that the earth is flat, and not spherical. The collective investigation conducted with so much pains and cost by the British Medical Association, has, among other subjects, gathered evidence on the relative longevity of different classes. It was stated a short time ago that the collective investigators, whose opportunities and labours have been almost world-wide, had decided that otal abstainers compared unfavourably with moderate drinkers, and even drunkards, with regard to longevity. Everybody was astonished, even the moderate drinkers themselves; and abstainers and life assurance offices declined to accept the supposed conclusion. A short time ago Dr. Owen, one of those who had been engaged in the investigation, took an opportunity of explaining the origin of the rumour, and also of exposing its fallacy. An idea had got abroad, said Dr. Owen, that total abstinence was a very bad thing, and that total abstainers had a relatively earlier mortality than drunkards. This he emphatically denied. The actual facts were these: The temperate had an average of 62 years of life, the intemperate of 52. Total abstainers, on the other hand, did not reach an average of more than 51 years. But this was shown to be clearly due to this one fact, and to it only: that total abstinence preponderates largely among young people, even among children, so that no proper comparison can be made on these data alone. When "all sorts and conditions" of men and women above 40 years of age were compared, it was found that the average expectation of life—in other words, the average longevity of abstainers—was four years in excess of the intemperate. The investigation also showed that the earlier death of the intemperate was caused in the majority of cases by distinctly alcholic diseases. There is nothing here to show whether abstainers or moderate drinkers live the longer; but the comparison between abstainers and intemperates is absolutely conclusive and indisputable. Temperance lecturers and assuran offices are proved to be entirely in the right.

